# Expression of vascular endothelial growth factor, matrix metalloproteinase-9 and E-cadherin in the process of lymph node metastasis in oesophageal cancer

**DOI:** 10.1038/sj.bjc.6690530

**Published:** 1999-07

**Authors:** F Sato, Y Shimada, G Watanabe, S Uchida, T Makino, M Imamura

**Affiliations:** Department of Surgery and Surgical Basic Science, Graduate School of Medicine, Kyoto University, Kawara-cho 54, Shogoin, Sakyo-ku, Kyoto, 606-8507, Japan

**Keywords:** oesophageal cancer, lymph node metastasis, MMP-9, VEGF, E-cadherin

## Abstract

Lymph node metastasis is a strong independent prognostic factor for oesophageal cancer. The expression of matrix metalloproteinases (MMPs) and reduction of E-cadherin correlate with lymph node metastasis of oesophageal cancer. We previously reported that the expression of vascular endothelial growth factor (VEGF) is associated with lymph node metastasis. This study was designed to determine whether VEGF, MMP-9 and E-cadherin expression is stable or changes in the process of lymph node metastasis of oesophageal cancer. Using immunohistochemistry, we detected VEGF, MMP-9 and E-cadherin expression in paraffin-embedded specimens of oesophageal squamous cell carcinoma. We classified 134 primary tumours and 174 nodal metastases using two different criteria: the absence [Group N(–)] or presence [Group N(+)] of nodal metastasis, and the stage of metastasis – Early Stage (cancer cells < 50% of lymph node) or Late Stage (≥ 50%) – and compared the expression among two groups and among two stages. The expression rates of Group N(–), Group N(+), Early Stage and Late Stage are as follows: VEGF (49%, 74%, 60%, 33%), MMP-9 (76%, 65%, 95%, 69%) and E-cadherin (49%, 24%, 55%, 38%). VEGF expression was down-regulated in Late Stage lymph node metastasis, while MMP-9 expression was elevated in Early Stage metastasis. E-cadherin expression is restored somewhat in Early Stage metastasis, but suppressed again in Late Stage metastasis. These data suggest that the expression of VEGF, MMP-9 and E-cadherin each change in the process of lymph node metastasis in oesophageal cancer, and that the patterns of change are different. © 1999 Cancer Research Campaign


*British Journal of Cancer* (1999) **80**(9), 1366–1372
© 1999 Cancer Research Campaign
Article no. bjoc.1998.0530

### Expression of vascular endothelial growth factor, matrix metalloproteinase-9 and E-cadherin in the process of lymph node metastasis in oesophageal cancer

**F Sato, Y Shimada, G Watanabe, S Uchida, T Makino and M Imamura**

Department of Surgery and Surgical Basic Science, Graduate School of Medicine, Kyoto University, Kawara-cho 54, Shogoin, Sakyo-ku, Kyoto, 606-8507, Japan

**Summary** Lymph node metastasis is a strong independent prognostic factor for oesophageal cancer. The expression of matrix metalloproteinases (MMPs) and reduction of E-cadherin correlate with lymph node metastasis of oesophageal cancer. We previously reported that the expression of vascular endothelial growth factor (VEGF) is associated with lymph node metastasis. This study was designed to determine whether VEGF, MMP-9 and E-cadherin expression is stable or changes in the process of lymph node metastasis of oesophageal cancer. Using immunohistochemistry, we detected VEGF, MMP-9 and E-cadherin expression in paraffin-embedded specimens of oesophageal squamous cell carcinoma. We classified 134 primary tumours and 174 nodal metastases using two different criteria: the absence [Group N(–)] or presence [Group N(+)] of nodal metastasis, and the stage of metastasis – Early Stage (cancer cells < 50% of lymph node) or Late Stage (≥ 50%) – and compared the expression among two groups and among two stages. The expression rates of Group N(–), Group N(+), Early Stage and Late Stage are as follows: VEGF (49%, 74%, 60%, 33%), MMP-9 (76%, 65%, 95%, 69%) and E-cadherin (49%, 24%, 55%, 38%). VEGF expression was down-regulated in Late Stage lymph node metastasis, while MMP-9 expression was elevated in Early Stage metastasis. E-cadherin expression is restored somewhat in Early Stage metastasis, but suppressed again in Late Stage metastasis. These data suggest that the expression of VEGF, MMP-9 and E-cadherin each change in the process of lymph node metastasis in oesophageal cancer, and that the patterns of change are different.

**Keywords:** oesophageal cancer; lymph node metastasis; MMP-9; VEGF, E-cadherin

The overall prognosis for oesophageal squamous cell carcinoma is still poor, with 5-year survival of 5–45% (Lerut et al, 1992; Ide et al, 1994; Roder et al, 1994). Some factors are thought to influence survival, such as sex, stage, microscopic grade, DNA ploidy, epidermal growth factor receptor (EGFR), p53 and lymph node metastasis (Rosai, 1996). Several studies using multivariate analysis indicated that lymph node metastasis is a strong independent prognostic factor (Theunissen et al, 1991; Roder et al, 1994; Tanigawa et al, 1997). We previously reported that the expression of vascular endothelial growth factor (VEGF) is associated with lymph node metastasis and prognosis in oesophageal cancer (Uchida et al, 1998). Other reports showed that the expression of matrix metalloproteinases (MMPs) (Shima et al, 1992), E-cadherin (Kadowaki et al, 1994; Nakanishi et al, 1997) and p53 (Wang et al, 1994) in primary tumours correlated with lymph node metastasis of oesophageal cancer.

From the aspect of therapy, angiogenesis-mediators and MMPs have been the recent focus of anti-cancer therapy (Gastl et al, 1997). If we are to use anti-angiogenesis drugs or MMP inhibitors as either adjuvant therapy after surgery or therapy for recurrent tumours, we will clearly require more information regarding the expression of angiogenesis-mediators and MMPs in metastatic tumours. However, few studies describe the expression of these proteins in lymph node metastasis. Therefore, the question remains: do cancer cells continue to express these factors in the lymph nodes?

*Received 14 July 1998*
*Revised 4 December 1998*
*Accepted 4 December 1998*

*Correspondence to:* Y Shimada

Previous investigators have described several steps in the metastatic process (Liotta, 1992; Chambers and Matrisian, 1997). In the process of lymph node metastasis, sequential steps after entry into lymph node would include the following: adhesion to the endothelium of the peripheral sinus, invasion to the parenchyma of the lymph node and sustained growth of cells in the lymph node. What is not yet known, however, is whether the expression of VEGF, MMPs and E-cadherin in cancer cells is changed during these steps of lymph node metastasis.

To answer these questions, we compared the immunohistochemical expression of VEGF, MMP-9, E-cadherin and p53, not only between primary tumours and lymph node metastases of oesophageal cancer, but also between the Early Stage and Late Stage of lymph node metastasis (for a definition of staging see Materials and Methods).

### MATERIALS AND METHODS

### Samples

We obtained 134 primary tumour samples from 134 patients with oesophageal squamous cell carcinomas from April 1984 to September 1996 treated in the Department of Surgery and Surgical Basic Science, Kyoto University. We divided 134 primary tumour samples into two groups. Group N(–) ($n$ = 37) were resected from patients without synchronous lymph node metastasis or nodal recurrence in follow-up period (≥ 2 years), and Group N(+)

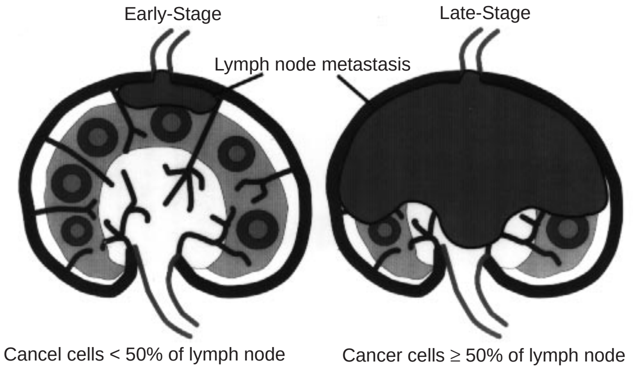

**Figure 1** A schema of the staging of lymph node metastasis. We decided on the stages of lymph node metastasis according to space occupation rate of cancer cells in the maximum cross section. When cancer cells occupied less than 50% of the lymph node, the sample was termed 'Early Stage'. When cancer cells occupied more than 50% of lymph node, it was termed 'Late Stage'

(*n* = 97) were from patients with lymph node metastasis, including one pN0 case with metachronous nodal metastasis.

We obtained 174 lymph node metastasis samples from 35 patients selected from Group N(+). We classified 174 lymph node metastases into two groups, according to the space occupation rate of cancer cells in the maximum cross-section of lymph node (Figure 1). There were 56 nodal metastases classified as Early Stage (cancer cells < 50% of lymph node) and 118 metastases classified as Late Stage (≥ 50%). To detect micro-metastasis within the lymph node and to estimate the distribution of cancer cells accurately, we used both haematoxylin and eosin (H&E) stain and anti-cytokeratin immunohistochemistry.

### Antibodies

We used five different antibodies, against VEGF, MMP-9, E-cadherin, p53 and cytokeratin. A rabbit polyclonal antibody to VEGF was a generous gift from Dr Ishiwata (Department of Joint Disease, Nippon Medical School, Tokyo, Japan) (Nagashima et al, 1995). A mouse monoclonal antibody (HECD-1) directed against E-cadherin was a kind gift from Dr Takeichi (Department of Biophysics, Faculty of Science, Kyoto University, Kyoto, Japan). This antibody was used as a 1:100 dilution of culture supernatant of the hybridoma clone described previously (Shimoyama et al, 1989). The three other mouse monoclonal antibodies were commercially available: anti-hMMP-9 (clone 56-2A4, Fuji Chemical Industries, Ltd, Takaoka, Japan), anti-p53 (clone DO-7, Dako A/S, Glostrup, Denmark), and anti-cytokeratin (clone AE1/AE3, Dako A/S, Glostrup, Denmark). The reliability of the three antibodies has been described elsewhere (Woodcock Mitchell et al, 1982; Vojtesek et al, 1992; Kawahara et al, 1993).

### Immunohistochemistry

The tissue samples were fixed in 10% buffered formalin and embedded in paraffin. Paraffin sections were cut at a thickness of 4-μm and attached to APS-coated glass slides. Sections were dewaxed, hydrated and treated using a high temperature antigen retrieval technique (microwave heating for VEGF and p53, and autoclave heating for MMP-9, E-cadherin and cytokeratin). The endogenous peroxide was blocked with 0.3% hydrogen peroxide

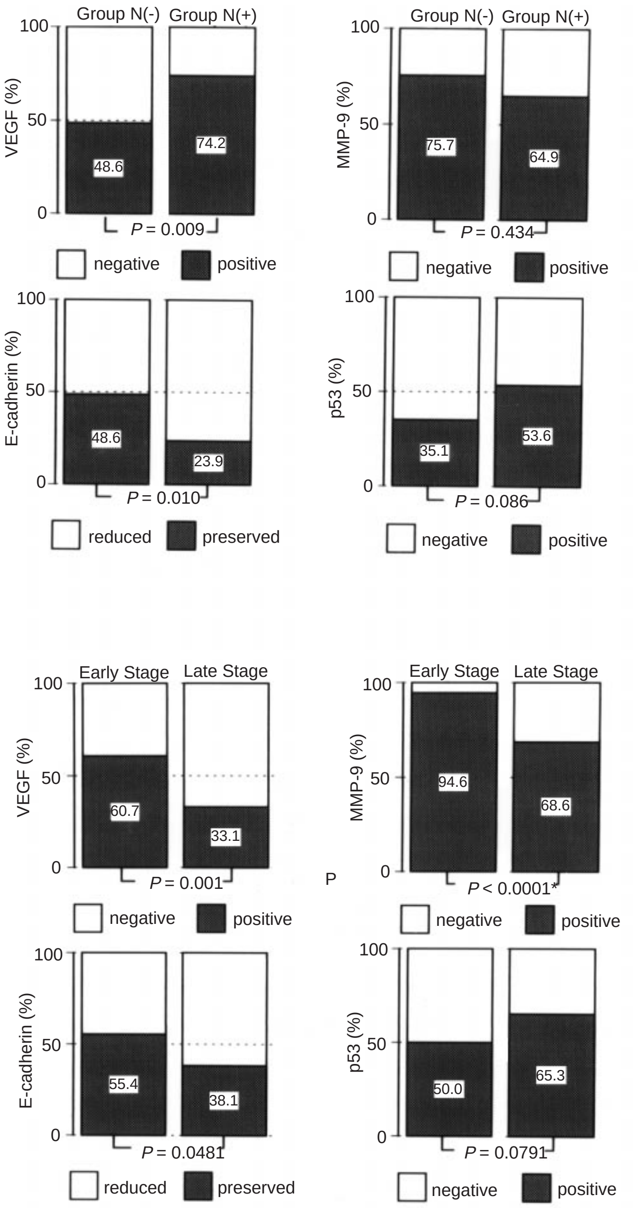

**Figure 2** The expression of VEGF, MMP-9, E-cadherin and p53 in primary tumour and lymph node metastasis. (**A**) The expression of Group N(–) (*n* = 37) and Group N(+) (*n* = 97) in primary tumour. (**B**) The expression in Early Stage (*n* = 56) and Late Stage (*n* = 118) of lymph node metastasis. *: Fisher's exact probability test in absolute methanol for 30 min. Thirty minutes of incubation with 10% normal horse serum or 10% normal goat serum eliminated non-specific staining. Excess normal serum was removed, replaced by the primary antibody overnight at 4°C. After washing the slides, sections were incubated with a 1:200 dilution of secondary antibody followed by avidin–biotin–peroxidase complex (Vecstain Elite ABC Kit, Vector Laboratories, Inc., Burlingame, CA, USA) for 40 min or 50 min respectively. Subsequently, sections were stained with 0.003% 3,3-diamino-benzidine tetrahydrochloride, 0.005% hydrogen peroxide in 0.05 M Tris–HCl buffer, pH 7.2, counterstained with Mayer's haematoxylin, dehydrated and mounted.

© 1999 Cancer Research Campaign

**Table 1** Clinicopathological background of patients with and without lymph node metastasis

| | Group N(–) | Group N(+) | Total | *P*-value |
|---|---|---|---|---|
| Gender | | | | |
| Male | 25 | 85 | 110 | *P* = 0.014 |
| Female | 12 | 12 | 24 | |
| Age | | | | |
| Range (years) | 46–84 | 39–84 | 39–84 | |
| Mean±s.d. | 65.2±10.1 | 62.7±10.0 | 63.4±10.0 | *P* = 0.202 |
| TNM classification | | | | |
| pTis | 2 | 0 | 2 | |
| pT1 | 20 | 13 | 33 | |
| pT2 | 6 | 23 | 29 | *P* < 0.0001 |
| pT3 | 5 | 43 | 48 | |
| pT4 | 4 | 18 | 22 | |
| pN0 | 37 | 1[a] | 38 | |
| pN1 | 0 | 96 | 96 | |
| pM0 | 37 | 59 | 96 | |
| pM1 | 0 | 38 | 38 | |
| | | (M1a; 16, M1b; 20) | | |
| Stage | | | | |
| 0 | 2 | 0 | 2 | |
| I | 20 | 0 | 20 | |
| IIa | 11 | 1 | 12 | |
| IIb | 0 | 21 | 21 | *P* < 0.0001 |
| III | 4 | 37 | 41 | |
| IV | 0 | 38 | 38 | |
| | | (IVa; 16, IVb; 20) | | |
| Differentiation grade[b] | | | | |
| GX | 3 | 0 | 3 | |
| G1 (well) | 8 | 13 | 21 | *P* = 0.383 |
| G2 (moderate) | 16 | 52 | 68 | |
| G3 (poor) | 10 | 32 | 42 | |

Group N(–); patients without synchronous lymph node metastasis or nodal recurrence in follow-up period (≥2 years), Group N(+), patients with lymph node metastasis, including one N0 case with metachronous nodal metastasis. [a]Group N(+) includes a N0 case with metachronous lymph node metastasis. [b]In three cases, grade of differentiation could not be assessed because almost all or all lesions were limited in epithelia (two; Tis, one; T1).

### Evaluation of immunohistochemical staining

VEGF staining was classified into two groups: negative and positive, as previously described (Uchida et al, 1998). Shima et al (1992) classified the stains of MMPs in oesophageal cancer into three groups. We, however, classified MMP-9 staining into two groups to simplify the data: negative, when the intensity of stain in cancer cells was negative or weaker than normal oesophageal epithelia; positive, when the intensity of staining was equal to or stronger than normal epithelia. According to estimates of positive-staining cell rates, E-cadherin and p53 were divided into two groups: preserved (≥ 50%) and reduced (< 50%), negative (< 10%) and positive (≥ 10%) respectively.

### Histology and staging

Histological evaluations were based on the TNM classification proposed by the International Union against Cancer in 1997 (UICC, 1997).

### Statistical analysis

Proportional analysis between sample groups was performed with the $\chi^2$ test with Yates' correction and Fisher's exact probability test in 2 × 2 contingency tables. All tests were two-sided, and results were considered significant when $P < 0.05$.

### RESULTS

### Expression of VEGF, MMP-9, E-cadherin and p53 in primary tumours

VEGF expression correlated with lymph node metastasis: 48.6% in Group N(–), 74.2% in Group N(+). E-cadherin expression was classified as reduced in 76.3% of Group N(+), compared with 51.4% of Group N(–). This demonstrates the relationship between reduction of E-cadherin in primary tumours and lymph node metastasis. With regard to MMP-9 expression in primary tumours,

**Table 2** Inter-nodal heterogeneity of VEGF, MMP-9, E-cadherin and p53

| | Early-stage (%) | Late-stage (%) | Overall (%) |
|---|---|---|---|
| VEGF | 8/15 (53.3) | 11/23 (47.8) | 17/28 (60.7) |
| MMP-9 | 2/15 (13.3) | 8/23 (34.8) | 12/28 (42.9) |
| E-cadherin | 7/15 (46.7) | 12/23 (52.2) | 16/28 (57.1) |
| p53 | 5/15 (33.3) | 5/23 (21.7) | 7/28 (25.0) |

*© 1999 Cancer Research Campaign*

we found no statistical difference between Group N(–) and N(+). The expression of p53 tended to be enhanced in Group N(+), but this was not significant (Figure 2).

### Expression of VEGF, MMP-9, E-cadherin and p53 in lymph node metastases

VEGF expression in lymph node metastasis is down-regulated with the development of the metastatic tumour. VEGF-positive staining decreased from 60.7% in Early Stage to 33.1% in Late Stage. MMP-9 expression was up-regulated only in Early Stage. The MMP-9-positive rate is elevated to 94.6% in Early Stage, and returns to 68.6% – similar to the level observed in primary tumour.

E-cadherin expression is restored in Early Stage, but suppressed again in Late Stage. The expression of p53 was enhanced in Late Stage relative to Early Stage, but this was not significant.

Employing anti-cytokeratin immunohistochemistry, we found micro-metastases in 17 lymph nodes, which had been diagnosed as normal by usual pathological examination using H&E staining. Among these metastases, we were able to evaluate the expression of VEGF, MMP-9, E-cadherin and p53 in nine lymph node metastases of seven patients: VEGF (7/9), MMP-9 (8/9), E-cadherin (5/9) and p53 (2/9). The expression of VEGF, MMP-9 and E-cadherin in micro-metastases were roughly consistent with that observed in Early Stage metastasis.

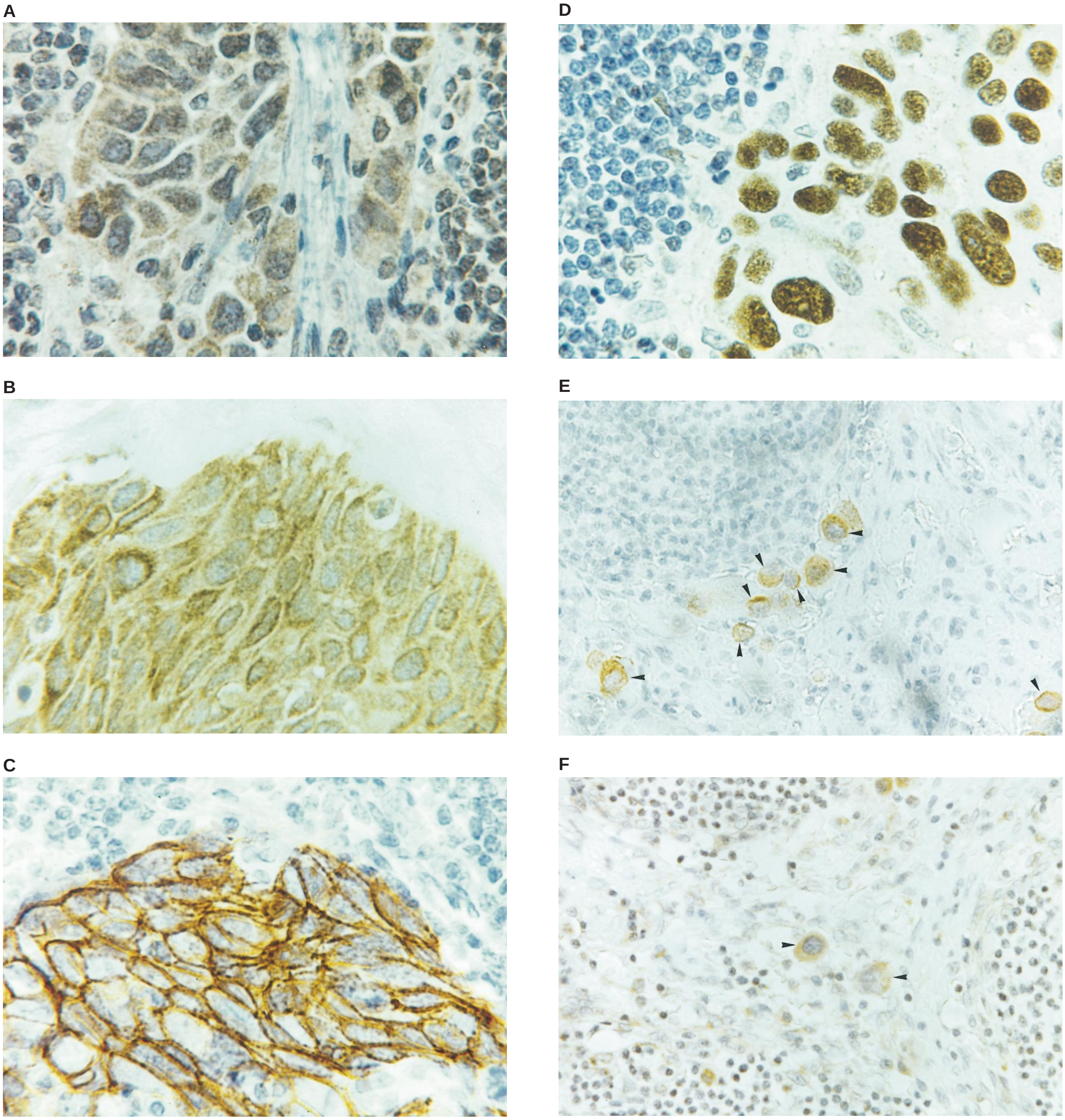

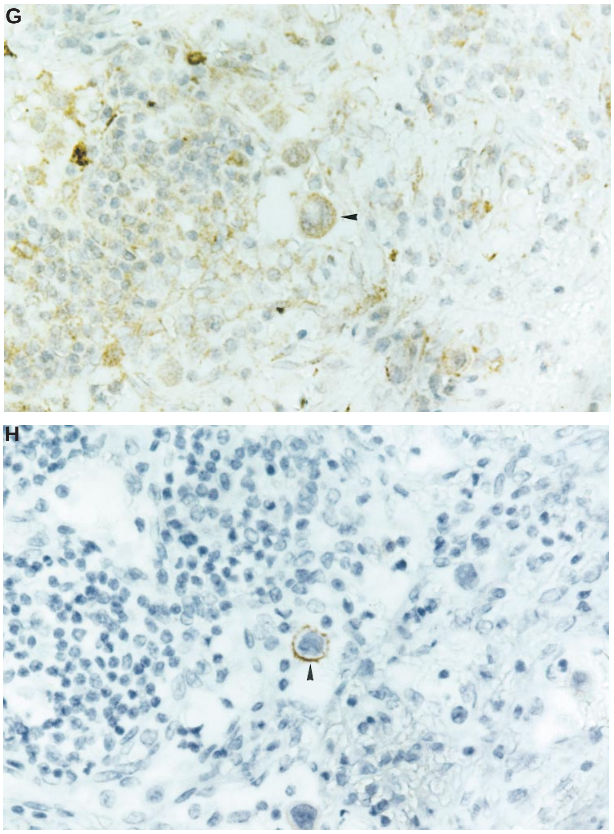

**Figure 3** Staining of VEGF, MMP-9, E-cadherin and p53 in lymph node metastasis. (**A–D**) Positive staining of VEGF, MMP-9, E-cadherin and p53 (**A**: VEGF, **B**: MMP-9, **C**: E-cadherin, **D**: p53). (**E–H**) Staining of micrometastasis in the same region (**E**: cytokeratin, **F**: VEGF, **G**: MMP-9, **H**: E-cadherin) (all: 200 ×). Arrowhead: stained cancer cells

### Clinicopathological background of patients

Patients studied consisted of 110 males and 24 females. The age of patients ranged from 39 to 84 (mean ± standard deviation (s.d.): 63.4 ± 10.0). Gender and pT-factor correlated with lymph node metastasis, but not differentiation grade or age (Table 1). With regards to the clinicopathological background and the expression of VEGF, MMP-9, E-cadherin and p53, we found no differences between patients selected from Group N(+) in the examination of lymph node and the others (data not shown).

### Localization of VEGF, MMP-9, E-cadherin and cytokeratin staining

VEGF stained the vesicle of cancer cells. Immunoreactivity against MMP-9 was found in the basal layers of normal oesophageal epithelia, macrophages, granulocytes, fibroblasts and the cytoplasm of cancer cells. E-cadherin expression was detected only in the cytomembrane, or both in the cytomembrane and the cytoplasm of cancer cells, and in the cytomembrane of normal epithelia.

This anti-cytokeratin antibody (AE1/AE3) recognizes many types of cytokeratin. Staining was detected in the cytoplasm of cancer cells and normal oesophageal epithelia. The staining of the cancer cells was intense and covered almost the entire cells in all but one of the 35 patients whose lymph nodes were examined.

### Heterogeneity between lymph node metastases

Frequently, patients with cancer have plural lymph node metastases. To ascertain whether all lymph node metastasis of a patient express equal amounts of VEGF, MMP-9, E-cadherin or p53, we compared the expression of these proteins among the metastases of each patient. The number of lymph node metastases obtained from the same patient ranged from 1 to 19 (mean ± s.d.: 4.74 ± 4.08). Fifteen patients had plural lymph node metastases in Early Stage, whereas 23 patients had Late Stage plural metastases.

Table 2 shows the number of these patients with inter-nodal heterogeneity of VEGF, MMP-9, E-cadherin and p53 expression (i.e. patients whose metastases varied with respect to the expression of these proteins). A high rate of overall inter-nodal heterogeneity is expected because the expression of VEGF, MMP-9 and E-cadherin changes from Early Stage to Late Stage. However, we found inter-nodal heterogeneity of expression of all four factors in each stage, ranging from 13.3% to 53.3%.

### DISCUSSION

The question addressed by the present study was whether the expression of VEGF, MMP-9, E-cadherin and p53 remains stable, or changes in the process of lymph node metastasis of oesophageal cancer. The main finding of this study is that the expression of VEGF, MMP-9, E-cadherin and p53 changes not only between the primary tumour and lymph node metastasis, but also between early- and late-stages of lymph node metastasis.

The pattern of change is different for each of these factors in the process of lymph node metastasis, and suggests where each factor might play an important role in the metastatic process. The pattern of VEGF is that VEGF expression of Group N(+) is much greater than that of Group N(–); however, the expression of VEGF is suppressed with the progression of metastatic stage. We and other investigators showed that over-expression of VEGF in primary tumours correlates with lymph node metastasis in oesophageal cancer and other tumours (Maeda et al, 1996; Moriyama et al, 1997; Ohta et al, 1997; Uchida et al, 1998). VEGF is a multifunctional molecule that has been implicated in vasculogenesis (Carmeliet et al, 1996; Ferrara et al, 1996), endothelial cell proliferation and migration (Senger et al, 1993), vascular permeability (Roberts and Palade, 1995), and stromal degradation through the activation of some proteolytic enzymes involved in tumour invasiveness and angiogenesis (Ferrara, 1996). However, it is not yet known which of VEGF's functions play an important role in promoting lymph node metastasis. On the other hand, it is generally believed that angiogenesis is required in secondary tumour progression (Folkman, 1990). Our data shows that in Early Stage metastasis VEGF is prominent when compared to Late Stage, and suggests that development in Late Stage tumour growth may be VEGF-independent, whereas the initial metastatic event may be VEGF-dependent or at least correlative.

The pattern of MMP-9 expression is that the MMP-9-positive rate is elevated in Early Stage of lymph node metastasis, whereas MMP-9 expression in primary tumour (both Group N(–) and N(+)) and Late Stage lymph node metastasis is lower. In our data, MMP-9 expression in primary tumour does not correlate with lymph node metastasis or any other clinicopathological factors (data not shown). The balance of activators and inhibitors, such as TIMP-1, regulates activity of MMP-9 (Liotta and Stetler Stevenson, 1991). In Early Stage metastatic lymph nodes, lymphocytes or macrophages

 *© 1999 Cancer Research Campaign*

surrounding cancer cells also produce MMP-9, and constitute a greater volume than do the metastatic cells. Therefore, it is unknown whether MMP-9 produced by cancer cells disturbs the balance between proteinases and inhibitors. However, our present findings suggest that cancer cells in Early Stage may react more strongly to a new microenvironment than cancer cells in Late Stage.

The pattern for E-cadherin is that the suppression of E-cadherin expression in primary tumour correlates with lymph node metastasis, and this result is compatible with findings of numerous other studies. In metastatic lesion, E-cadherin expression tends to be somewhat restored in Early Stage, but suppressed again in Late Stage. Cell dissociation and acquisition of cell motility are believed to affect the initial steps in the metastatic process. Therefore, suppressed expression or dysfunction of the cadherin–catenin complex might trigger the escape of cancer cells from the primary tumour. E-cadherin expression in metastatic lesion has been described by several authors, and became somewhat controversial (Schipper et al, 1991; Bongiorno et al, 1995; Jawhari et al, 1997; von Wasielewski et al, 1997). Shiozaki speculates that unstable E-cadherin reduction plays a role in terms of detachment and release from the primary lesion in the process of vessel invasion; however, when it comes to the establishment of actual metastasis, the opposite appears to be more favourable from the standpoint of attachment and growth as metastatic foci (Shiozaki et al, 1996). Our data also suggest that E-cadherin expression might offer some advantages in Early Stage of lymph node metastasis.

In this study, p53 expression was unchanged from Early Stage to Late Stage, whereas VEGF, MMP-9 and E-cadherin was down-regulated. These data indicate that the down-regulation of VEGF, MMP-9 and E-cadherin expression does not depend on non-specific reduction of overall protein synthesis of cancer cells. We previously reported the intimate relationship between VEGF expression and p53 mutation (Uchida et al, 1998). However, in this study, we found an inverse correlation between VEGF and p53 in lymph node metastasis. This finding suggests that VEGF would be regulated by pathways other than p53 in lymph node metastasis.

The mechanism of this regulation of VEGF, MMP-9, E-cadherin and p53 is unknown. Accumulation of gene abnormalities is generally thought to provide metastatic potential to cancer cells, and worsen prognosis (Vogelstein et al, 1989; Bland et al, 1995; Shimada et al, 1997). However, this study demonstrates that the acquired expression of malignant factors is not maintained through the end-stage of metastasis, but in fact changes. As the metastatic stage in the lymph node is advanced, the normal structure of the lymph node is gradually destroyed, and function of lymph nodes might be suppressed or lost. We suggest that the microenvironment (e.g. extracellular matrix, growth factors, cytokines and host defence) around cancer cells might change as cancer cells proliferate, and that as a consequence, the interaction between the cancer cells and the microenvironment might also change. In this study, we found inter-nodal heterogeneity as well as intra-nodal heterogeneity. Inter-nodal heterogeneity might be the result of not only heterogeneity of the cancer cell clones but also the alternative reaction of cancer cells to different microenvironments.

With regard to the staging of lymph node metastasis, Kurokawa (1970) divided lymph node metastases into three grades in an experimental model of lymph node metastasis. Shigetomi et al (1992) later modified Kurokawa's classification into four stages by observation of numerous serial sections, using the staging as an index of the metastatic potential (i.e. stage 0: cancer cells are limited in perinodal lymphatic vessels; stage 1: cancer cells are seen only in peripheral sinus; stage 2: cancer cells invade the cortex of lymph node, but not beyond 50%; stage 3: more than 50% of lymph node is occupied by cancer cell). However, to the best of our knowledge, no one has yet reported the correlation between the expression of cancer-related factors and the extent of lymph node metastasis. In order to simplify the data, we categorized the extent of lymph node metastases roughly into two stages by the space occupation rate of cancer cells (cut-off point: 50%).

From a therapeutic aspect, angiogenesis-mediators and MMPs have recently been the focus of targeted anticancer therapy (Gastl et al, 1997). If anti-angiogenesis drugs or MMP inhibitors are to be used as adjuvant therapy after surgery or therapy for recurrent tumours, information regarding the angiogenesis-mediators and MMPs in residual cancer cells will be required to judge the indication for the therapy. This study suggests that drugs directly inhibiting VEGF or MMPs might be more effective in micrometastasis or in cancer cells in Early Stage than in Late Stage.

In conclusion, the expression of VEGF, MMP-9 and E-cadherin are down-regulated from Early Stage to Late Stage of lymph node metastasis. Future analysis of the mechanisms responsible for these phenomena will be necessary.

### ACKNOWLEDGEMENTS

We thank Dr Hirohiko Yamabe, Laboratory of Anatomic Pathology, Kyoto University Hospital, for his kind assistance in histopathological technique, and Ms Ingrid Cuthbert for preparation of the manuscript. This work was supported in part by a Grant-in-Aid from the Japanese Ministry of Education, Science and Culture (Grant 09671301 and 09470265).

### REFERENCES

Bland KI, Konstadoulakis MM, Vezeridis MP and Wanebo HJ (1995) Oncogene protein co-expression. Value of Ha-*ras*, c-*myc*, c-*fos*, and p53 as prognostic discriminants for breast carcinoma. *Ann Surg* **221**: 706–720

Bongiorno PF, al Kasspooles M, Lee SW, Rachwal WJ, Moore JH, Whyte RI, Orringer MB and Beer DG (1995) E-cadherin expression in primary and metastatic thoracic neoplasms and in Barrett's oesophagus. *Br J Cancer* **71**: 166–172

Carmeliet P, Ferreira V, Breier G, Pollefeyt S, Kieckens L, Gertsenstein M, Fahrig M, Vandenhoeck A, Harpal K, Eberhardt C, Declercq C, Pawling J, Moons L, Collen D, Risau W and Nagy A (1996) Abnormal blood vessel development and lethality in embryos lacking a single VEGF allele. *Nature* **380**: 435–439

Chambers AF and Matrisian LM (1997) Changing views of the role of matrix metalloproteinases in metastasis. *J Natl Cancer Inst* **89**: 1260–1270

Ferrara N (1996) Vascular endothelial growth factor. *Eur-J-Cancer* **32a**: 2413–2422

Ferrara N, Carver Moore K, Chen H, Dowd M, Lu L, O'Shea KS, Powell Braxton L, Hillan KJ and Moore MW (1996) Heterozygous embryonic lethality induced by targeted inactivation of the VEGF gene. *Nature* **380**: 439–442

Folkman J (1990) What is the evidence that tumours are angiogenesis dependent? *J Natl Cancer Inst* **82**: 4–6

Gastl G, Hermann T, Steurer M, Zmija J, Gunsilius E, Unger C and Kraft A (1997) Angiogenesis as a target for tumour treatment. *Oncology* **54**: 177–184

Ide H, Nakamura T, Hayashi K, Endo T, Kobayashi A, Eguchi R and Hanyu F (1994) Esophageal squamous cell carcinoma: pathology and prognosis. *World J Surg* **18**: 321–330

Jawhari A, Jordan S, Poole S, Browne P, Pignatelli M and Farthing MJ (1997) Abnormal immunoreactivity of the E-cadherin–catenin complex in gastric carcinoma: relationship with patient survival. *Gastroenterology* **112**: 46–54

Kadowaki T, Shiozaki H, Inoue M, Tamura S, Oka H, Doki Y, Iihara K, Matsui S, Iwazawa T, Nagafuchi A. et al (1994) E-cadherin and α-catenin expression in human esophageal cancer. *Cancer Res* **54**: 291–296

Kawahara E, Okada Y, Nakanishi I, Iwata K, Kojima S, Kumagai S and Yamamoto E (1993) The expression of invasive behavior of differentiated squamous carcinoma cell line evaluated by an in vitro invasion model. *Jpn J Cancer Res* **84**: 409–418

Kurokawa Y (1970) Experiments on lymph node metastasis by intralymphatic inoculation of rat ascites tumor cells, with special reference to lodgement, passage, and growth of tumor cells in lymph nodes. *Gann* **61**: 461–471

Lerut T, De Leyn P, Coosemans W, Van Raemdonck D, Scheys I and LeSaffre E (1992) Surgical strategies in esophageal carcinoma with emphasis on radical lymphadenectomy. *Ann Surg* **216**: 583–590

Liotta LA (1992) Cancer cell invasion and metastasis. *Sci Am* **266**: 54–9, 62–63

Liotta LA and Stetler Stevenson WG (1991) Tumor invasion and metastasis: an imbalance of positive and negative regulation. *Cancer Res* **51**: 5054s–5059s

Maeda K, Chung YS, Ogawa Y, Takatsuka S, Kang SM, Ogawa M, Sawada T and Sowa M (1996) Prognostic value of vascular endothelial growth factor expression in gastric carcinoma. *Cancer* **77**: 858–863

Moriyama M, Kumagai S, Kawashiri S, Kojima K, Kakihara K and Yamamoto E (1997) Immunohistochemical study of tumour angiogenesis in oral squamous cell carcinoma. *Oral Oncol* **33**: 369–374

Nagashima M, Yoshino S, Ishiwata T and Asano G (1995) Role of vascular endothelial growth factor in angiogenesis of rheumatoid arthritis. *J Rheumatol* **22**: 1624–1630

Nakanishi Y, Ochiai A, Akimoto S, Kato H, Watanabe H, Tachimori Y, Yamamoto S and Hirohashi S (1997) Expression of E-cadherin, α-catenin, β-catenin and plakoglobin in esophageal carcinomas and its prognostic significance: immunohistochemical analysis of 96 lesions. *Oncology* **54**: 158–165

Ohta Y, Watanabe Y, Murakami S, Oda M, Hayashi Y, Nonomura A, Endo Y and Sasaki T (1997) Vascular endothelial growth factor and lymph node metastasis in primary lung cancer. *Br J Cancer* **76**: 1041–1045

Roberts WG and Palade GE (1995) Increased microvascular permeability and endothelial fenestration induced by vascular endothelial growth factor. *J Cell Sci* **108**: 2369–2379

Roder JD, Busch R, Stein HJ, Fink U and Siewert JR (1994) Ratio of invaded to removed lymph nodes as a predictor of survival in squamous cell carcinoma of the oesophagus. *Br J Surg* **81**: 410–413

Rosai J (1996) *Ackerman's Surgical Pathology*, Vol. 1. Mosby Year Book, Inc.: St Louis, MO.

Schipper JH, Frixen UH, Behrens J, Unger A, Jahnke K and Birchmeier W (1991) E-cadherin expression in squamous cell carcinomas of head and neck: inverse correlation with tumor dedifferentiation and lymph node metastasis. *Cancer Res* **51**: 6328–6337

Senger DR, Van de Water L, Brown LF, Nagy JA, Yeo KT, Yeo TK, Berse B, Jackman RW, Dvorak AM and Dvorak HF (1993) Vascular permeability factor (VPF, VEGF) in tumor biology. *Cancer Metastasis Rev* **12**: 303–324

Shigetomi A, Kotoh T, Harada T, Morikawa S and Nakamura T (1992) Development of an experimental model for spontaneous lymph node metastasis of human esophageal carcinoma in nude mice: histopathological analysis. *Hum Cell* **5**: 273–281

Shima I, Sasaguri Y, Kusukawa J, Yamana H, Fujita H, Kakegawa T and Morimatsu M (1992) Production of matrix metalloproteinase-2 and metalloproteinase-3 related to malignant behavior of esophageal carcinoma. A clinicopathologic study. *Cancer* **70**: 2747–2753

Shimada Y, Imamura M, Shibagaki I, Tanaka H, Miyahara T, Kato M and Ishizaki K (1997) Genetic alterations in patients with esophageal cancer with short- and long-term survival rates after curative esophagectomy. *Ann Surg* **226**: 162–168

Shimoyama Y, Hirohashi S, Hirano S, Noguchi M, Shimosato Y, Takeichi M and Abe O (1989) Cadherin cell-adhesion molecules in human epithelial tissues and carcinomas. *Cancer Res* **49**: 2128–2133

Shiozaki H, Oka H, Inoue M, Tamura S and Monden M (1996) E-cadherin mediated adhesion system in cancer cells. *Cancer* **77**: 1605–1613

Tanigawa N, Matsumura M, Amaya H, Kitaoka A, Shimomatsuya T, Lu C, Muraoka R and Tanaka T (1997) Tumor vascularity correlates with the prognosis of patients with esophageal squamous cell carcinoma. *Cancer* **79**: 220–225

Theunissen PH, Borchard F and Poortvliet DC (1991) Histopathological evaluation of oesophageal carcinoma: the significance of venous invasion. *Br J Surg* **78**: 930–932

Uchida S, Shimada Y, Watanabe G, Tanaka H, Shibagaki I, Miyahara T, Ishigami S, Arii S and Imamura M (1998) In oesophageal squamous cell carcinoma vascular endothelial growth factor is associated with p53 mutation, advanced stage and poor prognosis. *Br J Cancer* **77**: 1704–1709

UICC (1997) *TNM Classification of Malignant Tumours*, 5th edn. John Wiley: New York

Vogelstein B, Fearon ER, Kern SE, Hamilton SR, Preisinger AC, Nakamura and White R (1989) Allelotype of colorectal carcinomas. *Science* **244**: 207–211

Vojtesek B, Bartek J, Midgley CA and Lane DP (1992) An immunochemical analysis of the human nuclear phosphoprotein p53. New monoclonal antibodies and epitope mapping using recombinant p53. *J Immunol Methods* **151**: 237–244

von Wasielewski R, Rhein A, Werner M, Scheumann GF, Dralle H, Potter E, Brabant G and Georgii A (1997) Immunohistochemical detection of E-cadherin in differentiated thyroid carcinomas correlates with clinical outcome. *Cancer Res* **57**: 2501–2507

Wang DY, Xiang YY, Tanaka M, Li XR, Li JL, Shen Q, Sugimura, H and Kino I (1994) High prevalence of p53 protein overexpression in patients with esophageal cancer in Linxian, China and its relationship to progression and prognosis [published erratum appears in *Cancer* 1995: **75**: 1404]. *Cancer* **74**: 3089–3096

Woodcock Mitchell J, Eichner R, Nelson WG and Sun TT (1982) Immunolocalization of keratin polypeptides in human epidermis using monoclonal antibodies. *J Cell Biol* **95**: 580–588

*© 1999 Cancer Research Campaign*
